# Distribution and co-occurrence patterns of charophytes and angiosperms in the northern Baltic Sea

**DOI:** 10.1038/s41598-023-47176-8

**Published:** 2023-11-16

**Authors:** Kristjan Herkül, Kaire Torn, Tiia Möller-Raid, Georg Martin

**Affiliations:** Estonian Marine Institute, University of Tartu, Mäealuse 14, 12618 Tallinn, Estonia

**Keywords:** Plant sciences, Marine biology, Community ecology

## Abstract

The distribution data of 11 soft substrate charophyte and angiosperm species were analyzed. Our study aimed to elucidate the co-occurrence patterns among these sympatric macrophyte species and quantify their distribution areas. The central hypothesis of this study proposed that the observed co-occurrence patterns among the studied species deviate from what would be expected by random chance. Macrophyte occurrence data was derived from an extensive field sampling database. Environmental variables available as georeferenced raster layers including topographical, hydrodynamic, geological, physical, chemical, and biological variables were used as predictor variables in the random forest models to predict the spatial distribution of the species. Permutation tests revealed statistically significant deviations from random co-occurrence patterns. The analysis demonstrated that species tended to co-occur more frequently within their taxonomic groups (i.e., within charophytes and within angiosperms) than between these groups. The most extensive distribution overlap was observed between *Chara aspera* Willd. and *Chara canescens* Loisel., while *Zostera marina* L. exhibited the least overlap with the other species. The mean number of co-occurring species was the highest in *Chara baltica* (Hartman) Bruzelius while *Z. marina* had the largest share of single-species occurrences. Based on the distribution models, *Stuckenia pectinata* (L.) Börner had the largest distribution area.

## Introduction

Marine ecosystems are hard to access and thus there are many knowledge gaps, especially regarding biodiversity^1,2^. For example, in many cases we still lack spatially continuous data on the distribution area of marine benthic species though information on the benthic community structure, composition, and environmental variables has been collected over decades. In the marine environment the species distribution models (SDM) have gained attention and popularity since the 1990s with significant increase through time^3,4^; on the global scale the most studied groups have been fish, molluscs, and marine mammals^5^. A seamless map of distribution gives a significantly more relevant insight into species’ spatial patterns than a simple plotting of sampling sites on a map. Particularly, when considering that field sampling sites are usually spatially unequally distributed over extensive areas. Distribution patterns and areal estimations of benthic habitat formers are also a viable part of successful nature conservation and marine spatial planning and serve the aims of many related conventions and directives, e.g., Convention on biological diversity^6^, The Convention for the Protection of the Marine Environment in the Northeast Atlantic^7^, The Convention on the Protection of the Marine Environment of the Baltic Sea Area^8^, Council Directive 92/43/EEC of 21 May 1992 on the conservation of natural habitats and of wild fauna and flora, Directive 2008/56/EC of the European Parliament and of the Council of 17 June 2008 establishing a framework for community action in the field of marine environmental policy (Marine Strategy Framework Directive), Recommendation of the European Parliament and of the Council of 30 May 2002 concerning the implementation of Integrated Coastal Zone Management in Europe.

Marine environment is under a severe human pressure and coastal benthic communities are not an exception^9–11^. The shallow coastal waters are amongst the most productive ecosystems in the world and these habitats are also most affected by changes in oceanographic and atmospheric conditions and by various human impacts^12–16^. For the shallow soft-bottom marine habitats the presence of submerged aquatic vegetation is crucial as it serves various structural and functional roles e.g., benthic primary production, enhancing biodiversity, habitat provisioning, providing coastal protection, and stabilizing sediments^17,18^. Charophytes and angiosperms are the most characteristic species on the soft bottoms in the brackish Baltic Sea^19^; in total 23 species of charophytes and 91 species of angiosperms have been reported from this region^20^. Both monospecies and multispecies communities of charophytes and angiosperms are common^21–23^. Most of these species are of freshwater origin, somewhat adapted to brackish environments. Only *Zostera marina* L. represents the true marine species that is widespread also in lower salinity conditions^24,25^. Charophytes and eelgrass *Z. marina* are considered to be most important habitat formers in the soft bottom shallow coastal areas of the Baltic Sea^21,26^.

Decrease of submerged aquatic vegetation has been detected in both marine and freshwater ecosystems worldwide^27–30^. Charophytes are sensitive to pollutants and eutrophication and on the European scale have undergone a severe decline^31^. Similarly, in the submerged angiosperms group, seagrasses are affected by a wide variety of human activities and the accelerating loss of seagrass ecosystems across the globe is threatening coastal ecosystems^32,33^. Despite their significant ecological importance and declining distribution, research on the distribution and ecological preferences of submerged aquatic vegetation remains notably limited in comparison to studies on terrestrial vegetation^34–38^. More effort is needed to evaluate the status and trends of the benthic habitats and species in order to fully understand the mechanisms behind the changes^33^. However, the information on distribution patterns of soft bottom plant species is scarce and mostly in grey literature.

Set of abiotic environmental condition combined with biotic interactions, suitable for species to maintain the stable conditions in habitat, define the niche of the species^39,40^. Niche width determines habitat specialization which may indicate the species vulnerability to global changes, as worldwide decline in specialist species has been noticed^41^. Knowledge about species specialization enables a better understanding of the mechanisms and consequences of environmental change, especially in brackish and estuarine environments where species live close to their physiological limits^42^. It is known that spatially and temporally variable salinity, underwater light environment, and availability of suitable substrate are the main drivers of submerged vegetation in estuaries and brackish water ecosystems^19,43,44^. The distinct habitat preferences of charophytes and angiosperms along environmental gradients have been previously described by Herkül et al.^45^. Although the abundance of charophytes and angiosperms increase proportionally with increase of soft substrate^19^, the distributions of individual species along the salinity gradient are dependent on species specific salinity tolerance^43,45^. Previously, analysis of the niche breadth of the submerged soft-bottom macrophyte species showed a substantial variation of the niche breadths among the species. Furthermore, the angiosperms showed higher species-specific variability of niche breadths compared to those of the charophytes^45^. Most of the work so far has been done on species level e.g.,^46,47^, but studies comparing multidimensional niche overlaps of soft-bottom submersed vegetation are lacking. Further investigations involving the geographical aspects, for instance the species’ distribution areas and co-occurrence patterns, are needed to gain a more complete understanding of the patterns and the mechanisms of the regional coexistence of aquatic macrophytes. To date, only a limited number of studies have explored the co-occurrence of aquatic macrophytes while examining the hypothesis of non-random patterns. However, these studies focus on freshwater systems and make inferences at the community level as a whole, e.g.,^48–50^, rather than delving into species-specific pairwise patterns. There are only a few studies that investigate macrophytes to assess whether pairwise co-occurrence deviates from random patterns^51,52^ but these are also from freshwater environments. This study aims to address the research gap concerning co-occurrence patterns of soft substrate macrophytes within a brackish water environment. We highlight the importance of conducting pair-wise co-occurrence analysis as it serves as a fundamental step towards deducing the potential inter-specific mechanisms influencing the observed co-occurrence patterns.

This study focuses on spatial distribution patterns of characteristic plant species of soft-bottom habitat in the northern Baltic Sea region. Our central hypothesis suggested that the observed co-occurrence patterns among the studied species deviate from those expected by random chance. More specifically, our objectives were: (1) to provide an overview of the geographic distribution of angiosperms and charophytes in the Estonian sea area, northern Baltic Sea; (2) to elucidate the co-occurrence patterns of the studied charophytes and angiosperms, both within and between these groups. To address these objectives, we employed an extensive database of field inventories and species distribution modeling approach^53^. The use of species distribution modeling was crucial for transitioning from site-specific data to spatially continuous datasets. This transition, in turn, facilitated the creation of species distribution maps and the quantification of species distribution areas.

## Methods

### Study area

**Figure 1:**
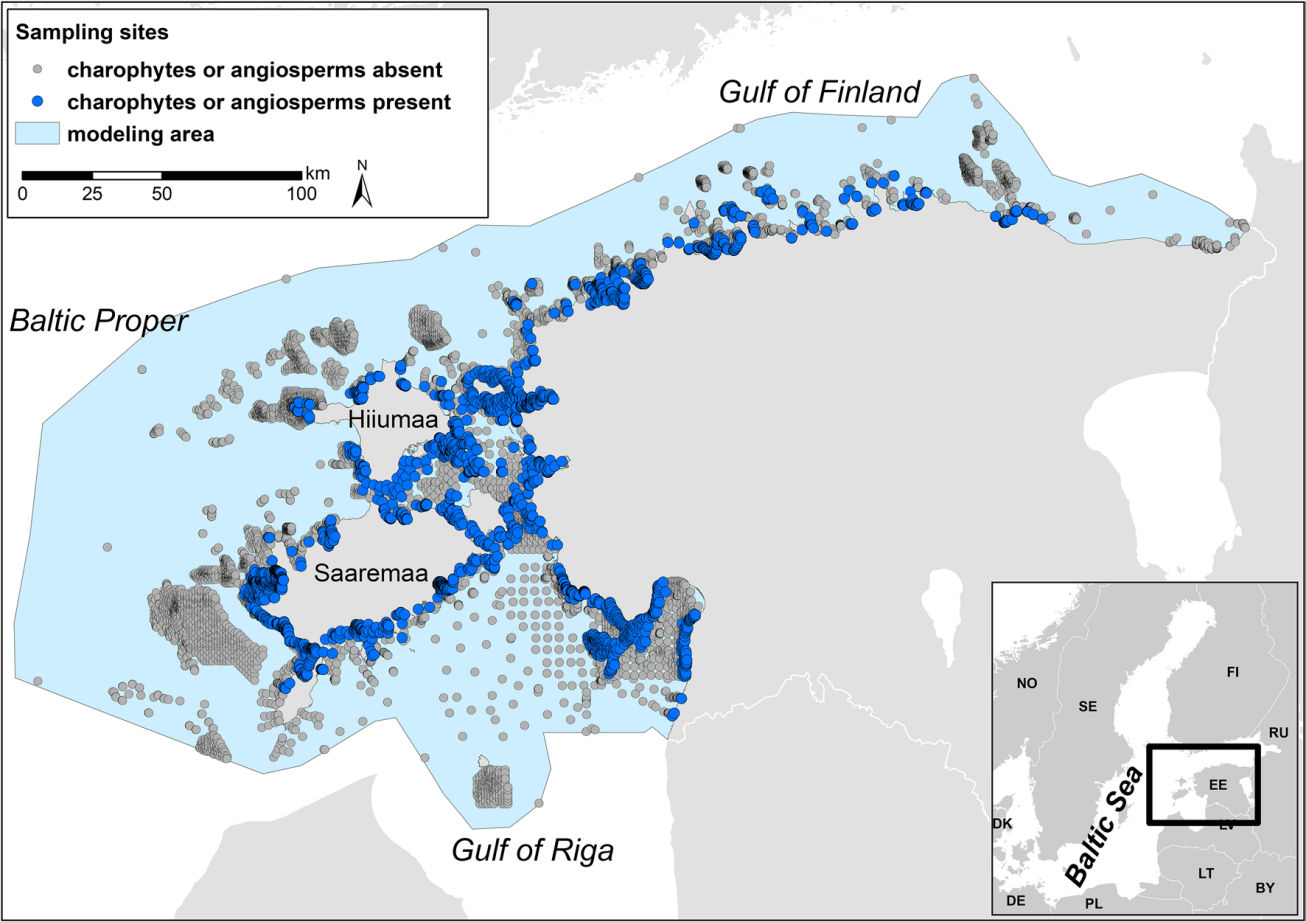
Study area and macrophyte sampling sites. The modeling area covered the full extent of the Estonian marine waters from the coastline to the outer border of the exclusive economic zone. *ESRI ArcMap* version 10.8.1 (https://esri.com) was used to generate this map.

The Baltic Sea is a tideless brackish water body. The phytobenthos data for this study originated from the Estonian marine area, northern Baltic Sea (Fig. 1). The study area included the full extent of the Estonian marine area from the coastline to the outer border of the exclusive economic zone (36,800 km^2^). The study area includes three large sub-basins of the Baltic Sea: The Baltic Proper, the Gulf of Finland, and the Gulf of Riga. The western study area around islands Saaremaa and Hiiumaa and the Gulf of Riga is characterized by extensive shallow coastal zone with numerous islands, islets, bays, and peninsulas. Contrastingly, the shallow coastal zone is limited due to steep coastal slope in the Gulf of Finland. Strong gradients of water depth, salinity and wave exposure exist in all sub-basins. The westernmost region of the study area is exposed to the open Baltic Proper with a wave fetch of hundreds of kilometers while the inner reaches of bays of the mainland are very sheltered, both by the mainland and by the islands. Likewise, the salinity gradient generally follows an east–west direction: salinity reaches seven in the Baltic Proper, while it falls to almost zero in the inner parts of the bays with riverine inflow. Hard limestone bedrock and granite boulders prevail in the most exposed areas. Mixed sediments of sand, gravel, and pebbles are typical in the mid-range of the wave exposure gradient. Fine sand and mud dominate in the most sheltered bays.

The abiotic environmental gradients drive the structure of macrophyte communities in the study area. Species of marine origin are common in the areas of the highest salinity, while freshwater species dominate in coastal areas with strong freshwater inflow. The brown alga *Fucus vesiculosus* L. and the red alga *Furcellaria lumbricalis* (Hudson) J.V. Lamouroux are the most important perennial habitat-forming algal species on hard substrate. Annual and perennial filamentous green, brown, and red algae like *Ulva intestinalis* L., *Cladophora glomerata* (Linnaeus) Kützing, *Pylaiella littoralis* (Linnaeus) Kjellman, *Ceramium tenuicorne* (Kützing) Waern, *Vertebrata fucoides* (Hudson) Kuntze are common on hard substrate. Several species of angiosperm plants are common on soft substrate, e.g., *Zostera marina* L., *Stuckenia pectinata* (L.) Börner, *Potamogeton perfoliatus* L., *Zannichellia palustris* L., *Myriophyllum spicatum* L. and *Ruppia maritima* L. The charophytes (*Chara* spp. L., *Tolypella nidifica* (O.F.Müll.) Leonh.) are common on sandy and muddy sediments in shallow waters of sheltered bays. As a result of eutrophication extensive growth of annual filamentous algae and formation of drift algal mats are common phenomena in the study area^54^.

### Biological sampling

Data for the study originated from a macrobenthos database of the Estonian Marine Institute, University of Tartu. The database stores an extensive amount of macrobenthos data starting from 1950s and including data from more than 55,000 benthic samples. The database incorporates georeferenced benthos data originating from various projects like basic scientific research, national marine monitoring, benthic habitat mapping studies, surveys related to environmental impact assessments etc. Data from the database was filtered to include only years 2005–2020 because samples from that period represent methodologically coherent dataset with good spatial coverage. Both biomass and percentage coverage data were used in this study. The quantitative biomass samples were collected using Ekman and Van Veen type bottom grab samplers or by scuba divers, who collected all the flora and fauna inside a 0.04 m^2^ metal frame. The samples were stored deep frozen (−18 °C) until analysis. In a laboratory, the samples were sorted and all macrobenthic organisms were identified under a microscope. Dry weights of all taxa were measured after two weeks drying at 60 °C. Biomasses were calculated per square meter. Data from replicate biomass samples (n = 3) of each sampling station were averaged. Coverage estimates were done either by scuba divers who assessed the percentage cover of all plant species or by using the so-called drop-camera, which was let down above the bottom of the sea by a cable. Videos were later analyzed on computer screen by estimating the average percentage coverage of substrate types and coverage of benthic macrophyte species seen in a video recording. Both video estimates and in situ estimates by scuba divers covered an area of about 2 m radius. Data from visual estimates were used only if visual information was sufficient for species level determination or the species determinations were confirmed by biomass samples. For the purposes of this study, the biomass and percentage cover data of macrophytes was transformed into binary occurrence data (0—absence, 1—presence). Specifically, if a species was detected in a sample, it was recorded as a presence, irrespective of the species’ quantity (cover or biomass). Conversely, if a species was not detected, it was recorded as an absence.

As the aim of the study was to address the geographical distribution of macrophytes, the temporal replicates from sampling sites, that were visited several times, were removed and only the data from the most recent sampling event were retained in the dataset. To eliminate spatial redundancy in the data, we applied a 25 m equirectangular grid to the study area. In cases where multiple observations fell within the same grid cell, we retained the data from the sampling site with the most recent year of data collection. If there were multiple sampling occasions in the most recent year, we randomly selected one sample for inclusion in the dataset. The application of these temporal and spatial filters resulted in data from 14,356 unique field sites (Fig. 1), with a total of 20 charophyte and angiosperm species. The species with a very low occurrence rates (< 100 sites) were excluded from the dataset to ensure comparable analytical results. Altogether 11 species were included in this study (Table 1).

**Table 1:** List of the studied species. The environmental information (B—brackish water, F—fresh water) is given based on Estonian data^55,56^.

Species	Clade	Environment	Salinity range
*Myriophyllum spicatum* L.	Angiospermae	B/F	0–10^44^
*Potamogeton perfoliatus* L.	Angiospermae	B/F	0–12^45^
*Ruppia maritima* L.	Angiospermae	B	0–55 (65)^46^
*Stuckenia pectinata* (L.) Börner	Angiospermae	B/F	0–19^47^
*Zannichellia palustris* L.	Angiospermae	B/F	0–20^48^
*Zostera marina* L.	Angiospermae	B	(2.5)5–35(40)^24^
*Chara aspera* Willd.	Charophyta	B/F	0–15 (20)^49,50^
*Chara baltica* (Hartman) Bruzelius	Charophyta	B	2–10 (18)^51^
*Chara canescens* Loisel	Charophyta	B	1.5–21^52^
*Chara connivens* Salzm. ex A. Braun	Charophyta	B/F	0–9^28^
*Tolypella nidifica* (O.F.Müll.) Leonh.	Charophyta	B	(0)4–24^53^

### Environmental variables

Altogether 14 environmental variables including topographical (depth, slope of seabed), hydrodynamic (wave exposure, currents, ice conditions), geological (seabed substrate), physico-chemical (temperature, salinity, transparency, nutrients), and biological (water chlorophyll concentration) variables were used in this study (Table 2, Appendix 1). Long-term mean values were used for variables with time series, because general spatial distribution patterns not temporal variation (seasonal, annual) of macrophytes was addressed in this study. All environmental variables were available as georeferenced raster layers in GeoTIFF format. The extents of raster layers fully covered the study area. Raster cell size was 10 m for depth and seabed slope and 50 m for all the other variables. The environmental variables were selected based on previous knowledge on potential relationships with the distribution of the studied macrophytes, e.g.^45,55^, and data availability.

**Table 2:** Environmental variables. See Appendix 1 for schematic overview maps of the general spatial patterns of the variables.

Variable	Abbreviation	Source
Water depth (m)	depth	1
Mean water depth in 2 km radius (m)	depth_2km	1
Slope of seabed (°)	slope	1
Slope of seabed in 2 km radius (°)	slope_2km	1
Salinity (PSU): long-term (2002–2008) mean	salinity	2,3
Wave exposure based on simplified wave model (m^2^ s^−1^): based on long-term (2003–2019) mean wind speeds and directions	wave	4
Current velocity (m s^−1^): long-term (2005–2019) mean	current	5
Water temperature in warm season (°C): long-term (2005–2019) mean in May–October	temp_warm	5
Water temperature in cold season (°C): long-term (2005–2019) mean in November–April	temp_cold	5
Concentration of nitrates (mmol m^−3^): long-term (2005–2019) mean values in winter (December-February)	nitrate	6
Concentration of phosphates (mmol m^−3^): long-term (2005–2019) mean values in winter (December-February)	phosphate	6
Chlorophyll a content of sea surface (mg m^−3^): long-term (2005–2019) mean	chl	6
Proportion of ice cover (0…1): long-term (2000–2016) mean	ice	7
Proportion of soft sediment (0…1)	sediment	2

Sources:

1—Bathymetric data by Estonian Maritime Administration (georeferenced depth raster with 10 m pixel size).

2—Databases of the Estonian Marine Institute, University of Tartu.

3—COHERENS ocean circulation model^57^.

4—Simplified wave model based of fetch and wind data^58,59^.

5—Copernicus Marine Environment Monitoring Service’s Baltic Sea Physics Reanalysis product BALTICSEA_REANALYSIS_PHY_003_011 (https://resources.marine.copernicus.eu/product-detail/BALTICSEA_REANALYSIS_PHY_003_011).

6—Copernicus Marine Environment Monitoring Service’s Baltic Sea Biogeochemical Reanalysis product BALTICSEA_REANALYSIS_BIO_003_012 (https://resources.marine.copernicus.eu/product-detail/BALTICSEA_REANALYSIS_BIO_003_012).

7—Ice data developed by the Marine Systems Institute, Tallinn University of Technology^60^.

### Data analysis

*R* programming language version 4.2.2^61^ in the development environment *RStudio*^62^ was used for data preparation, analysis, and modeling. In addition to the base *R* functionality, the following packages were used: *raster*^63^ for handling spatial raster data, *sf*^64^ for handling spatial vector data, *randomForest*^65^ for fitting random forest models, *caret*^66^ for creating folds for cross-validation and for calculating Kappa coefficients, *PresenceAbsence*^67^ for converting probability of occurrence to presence-absence, *tidyverse*^68^ for tabular data manipulation and plotting. *ArcMap* software^69^ was used to generate the map of the study area.

Species distribution modeling (SDM) framework^53^ was used to generate seamless distribution maps of the studied macrophyte species. Random forest (RF) was chosen as the modeling method because it offers many advantages over alternative methods: a) RF has proven to be one of the most accurate methods in SDM; b) RF boasts a minimal number of user-settable parameters, with default settings consistently producing reliable results; c) random selection of predictor variables at each node mitigates the risk of overfitting; d) RF excels at directly handling the modeling of binary, continuous, and nominal variables^65,70–79^. RF is a machine learning method that generates a large number of regression trees, each calibrated on a bootstrap sample of the original data^58^. Each node is split using a subset of randomly selected predictors and the tree is grown to the largest possible extent without pruning. RF can handle both regression and classification type of modeling. For predicting a value of a new data point, the data are run through each of the tree in the forest and each tree provides a value. The model prediction is then calculated as the average value over the predictions of all the trees in the forest in regression model and by majority vote in classification model.

The species presence-absence data from the 14,356 field sampling sites (Fig. 1; see the section Biological sampling) was used as a response variable in RF models. We employed both regression-type and classification-type RF models as candidate models for each species. The motivation for fitting both regression and classification models was to identify the optimal RF model type based on their performance characteristics. The values of the 14 environmental variables (Table 1; see section Environmental variables) served as predictor variables. A separate model was built for each macrophyte species. The models were then used to generate spatial distribution prediction for each species. Using the trained RF models, species’ predictions were calculated for each data point in the prediction dataset (n = 3,685,338) covering the whole Estonian marine area from the coastline to the outer border of the exclusive economic zone (36,800 km^2^) with 100 m rectangular equispaced grid (see modeling area in Fig. 1). The values of all environmental variables (predictor variables sensu predictive modeling) were available for each point in the prediction dataset. Given the binary nature of the input data (0 for absence, 1 for presence), the RF regression-type model generated probability estimates of occurrence as predictions, falling within the range of zero to one (encompassing any decimal value between zero and one). Conversely, in the classification-type RF model, the prediction outcome was a binary classification, with values of zero and one. The predictions of regression-type models (probabilities of occurrence) of each species were separately converted to binary presence-absence classes (1—presence, 0—absence) using the sensitivity–specificity difference minimizer method that has been found to produce more accurate results compared to other methods^80–82^. The conversion of regression-type RF outcome to binary presence-absence was done in order to estimate the distribution area of the species and to compare the predictive performance with that of classification-type RF outcome.

In RF model fitting, the function *randomForest* in package *randomForest* was used. Two parameters must be set in *randomForest*: the number of predictor variables to be randomly selected at each node (*mtry*) and the number of trees in a forest (*ntree*). The *mtry* parameter was retained at its default values: one-third of the number of predictor variables for regression-type RF and the square root of the number of predictor variables for classification-type RF models, in accordance with the recommendations provided by Liaw and Wiener^65^. *ntree* was set to 1000 as already 500 trees usually yield stable results^65^. In all models, we verified the adequacy of using 1000 trees to achieve stable results by employing the *plot* function within the *randomForest* package. The importance of predictor variables was calculated using the permutation test inside the *randomForest* function by specifying *importance* = TRUE and *nPerm* = 10. All other arguments in the *randomForest* function were left as default values. Further information on the *R* code is shown in Appendix 2.

The predictive performance of the models was evaluated using tenfold cross-validation (CV)^83,84^. Using CV, area under the receiver operating curve (AUC)^85^ and Cohen’s kappa^86,87^ were calculated for regression-type RF models and only Cohen’s kappa for classification-type models. For calculating kappa in regression-type models, the probability of occurrence was converted into binary classes (1—presence, 0—absence) using the sensitivity–specificity difference minimizer method^82^. Stratified random sampling without replacement was used to generate data folds for each species using the presence-absence of a given species as strata to ensure equal proportions of presences in all folds^83^. Using ten folds resulted in a division of 90% data for model training and 10% of data for validation at each iteration. Tenfold cross-validation was done only to estimate the predictive performance of models. Final models for generating the spatial predictions were trained using 100% of the data. Mean AUC and kappa values over ten folds were used as the estimate of predictive performance. AUC values vary between 0.5 and 1. AUC value of 0.5 indicates that a model performs the same as random chance while the value of 1 indicates perfect model prediction^85,88^. There is no universal evaluation criteria of AUC values between 0.5 and 1, but often the values 0.5–0.6 are considered “poor”, 0.6–0.7 “fair”, 0.7–0.8 “good”, 0.8–0.9 “very good”, 0.9–1 “excellent”^74,89^. Following Landis and Koch^87^, the Cohen’s kappa coefficient value below zero indicates “poor”, 0–0.2 “slight”, 0.21–0.4 “fair”, 0.41–0.6 “moderate”, 0.61–0.8 “substantial” and 0.81–1 “almost perfect” strength of agreement.

Relationships between species richness (number of species) and the environmental variables were depicted using generalized additive models (GAM)^90^ with cubic regression splines. The degrees of freedom were limited to five in GAMs to avoid overfitting. Separate GAM fits were produced for each species richness–environmental variable pair for plotting purposes.

Two different methods were applied to assess the distribution overlap among the studied species: Schoener’s D index and proportion of co-occurrence in sampling sites. Species occurrences in the field data was used as the input for both methods. Schoener’s D can vary between zero and one: zero indicates no distribution overlap while one indicates full distribution overlap (i.e. identical distributions) between species. While there are several metrics available for estimating distribution overlap, Schoener’s D was chosen due to its good performance, simplicity, and long history of use^91,92^. While Schoener’s D enables to generate a single value for estimating the spatial overlap of each pair of the studied species, it cannot give full insight into the overlap patterns because a distribution overlap between two species is seldom symmetrical and the sizes of distribution areas vary. For example, the Schoener’s D value of 0.5 may mean that the distribution area of a species A is fully (100%) inside the distribution area of a species B while only 50% of the distribution of the species B coincides with that of A. This is possible given that the distribution area of B is twice the size of A. To account for the asymmetry, for each species, percentage overlap of distribution with all the other species was also calculated. Permutation test^93^ with 100,000 permutations was conducted for each pair of species to assess whether their co-occurrence, as measured by the Schoener's D value, deviated significantly from what would be expected by random chance^94^. By randomly shuffling the field sites and calculating the Schoener’s D based on the 100,000 iterations, the so called null distribution^95^ of D values for each pair of species was produced. The permutationally generated null distribution of D values was subsequently compared to the observed D value. The null hypothesis of random co-occurrence was rejected when the observed D value fell outside a predefined percentile range (e.g., 2.5% to 97.5% with a p-value threshold set at 0.05), signifying a substantial departure from random chance^93^. To account for multiple comparisons, the p-values were corrected using the Benjamini–Hochberg method^96^. R code for the permutation test is shown in Appendix 2. Kruskal–Wallis test^97^ was used to assess statistical differences of overlap both within and between the species groups based on the pair-wise D values with the following group levels: within charophytes, within angiosperms, between charophytes and angiosperms.

### Research involving human and/or animal participants

The study did not involve animal or human participants.

## Results

Based on the Cohen’s kappa coefficients of tenfold cross-validation, the average prediction accuracy of regression-type RF models was marginally higher than that of classification-type models (Table 3) and subsequently the regression-type RF models were selected to produce the maps of species distribution. The AUC values of all species were over 0.89 indicating high prediction accuracy while the model performance measured using kappa coefficients indicated mainly fair to moderate agreement between predicted and observed species occurrences (Table 3). Notably low Cohen’s kappa coefficient of 0.15 was found in *T. nidifica*. Depth was the most influential environmental variable in the regression-type RF models, followed by proportion of soft sediment and slope of seabed (Appendix 3).

**Table 3:** Results of tenfold cross-validation of regression-type and classification-type RF models. Average values of the statistics over all species are shown in the last row. AUC—area under the receiver operating curve, kappa—Cohen’s kappa coefficient.

Species	Regression RF		Classification RF
	AUC	kappa	kappa
*C. aspera*	0.963	0.529	0.498
*C. baltica*	0.943	0.274	0.213
*C. canescens*	0.95	0.36	0.288
*C. connivens*	0.947	0.275	0.236
*T. nidifica*	0.894	0.156	0.127
*M. spicatum*	0.948	0.507	0.469
*P. perfoliatus*	0.955	0.462	0.424
*R. maritima*	0.926	0.348	0.309
*S. pectinata*	0.948	0.645	0.632
*Z. palustris*	0.901	0.323	0.285
*Z. marina*	0.943	0.427	0.351
Average	0.938	0.391	0.348

**Figure 2:**
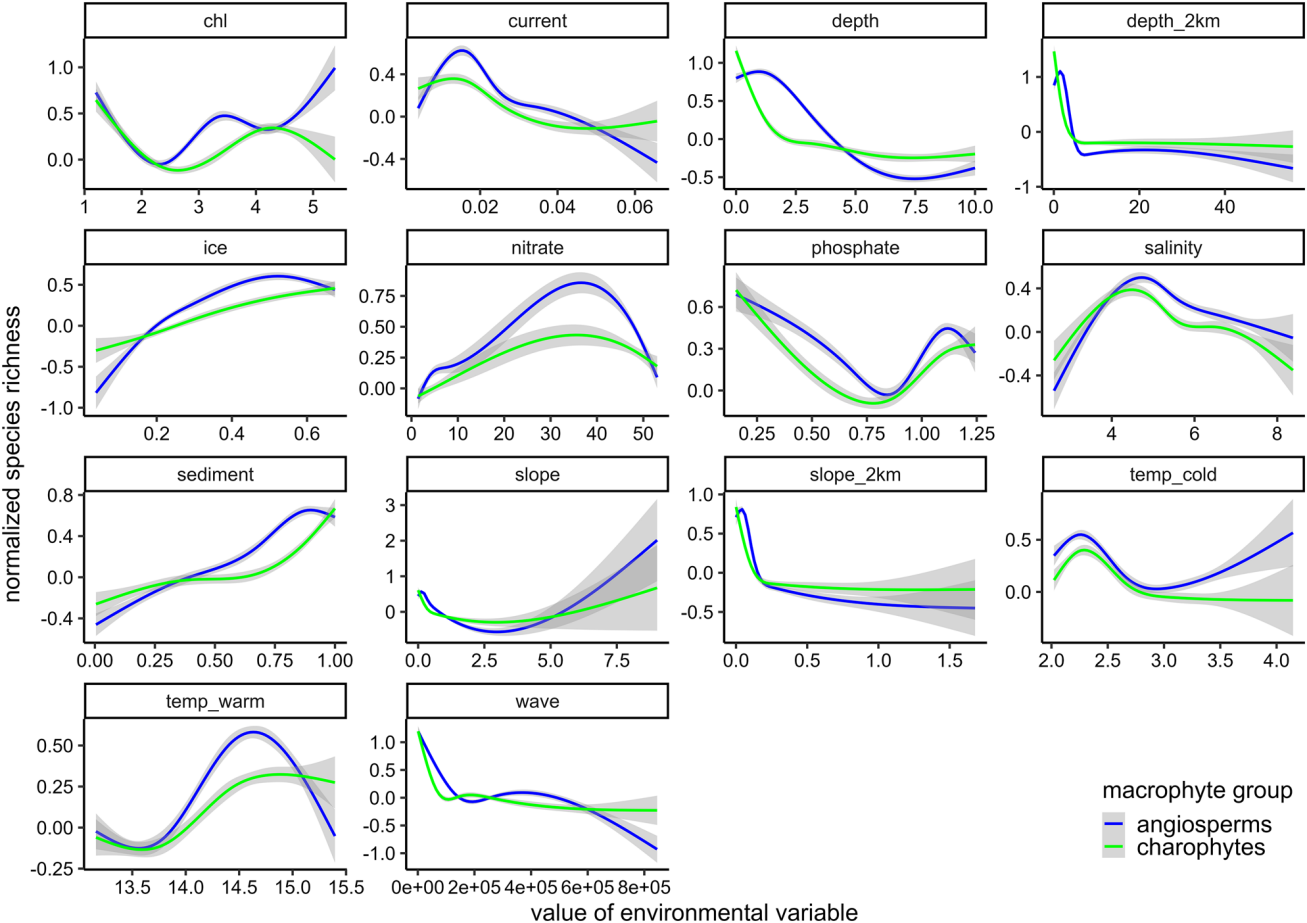
Variability of the species richness of angiosperms and charophytes along the environmental gradients as fit by GAMs. 95% confidence interval is shown in gray. Data from sampling sites with water depth exceeding 10 m is excluded.

The species richness of charophytes sharply decreased with increasing depth while the richness of angiosperms exhibited the highest values between 0 to 2 m. However, generally, the species richness of both angiosperms and charophytes followed similar patterns along the environmental gradients (Fig. 2).

**Figure 3:**
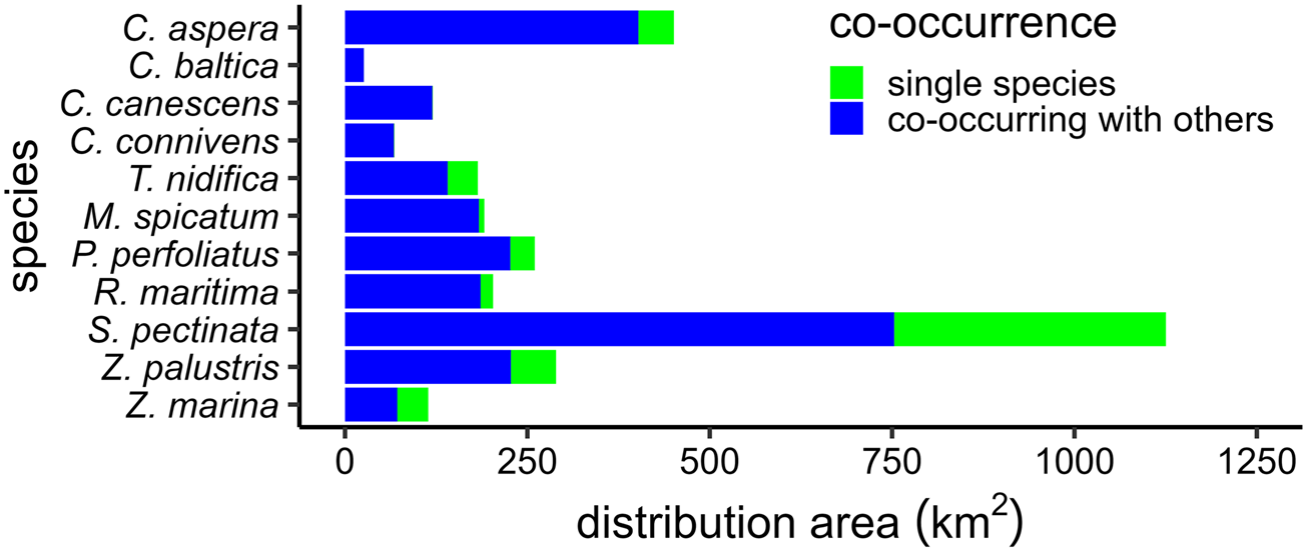
Distribution area of the studied species based on distribution modeling. Colors indicate the distribution area of a species occurring alone (single species) or together with at least one other studied species.

**Figure 4:**
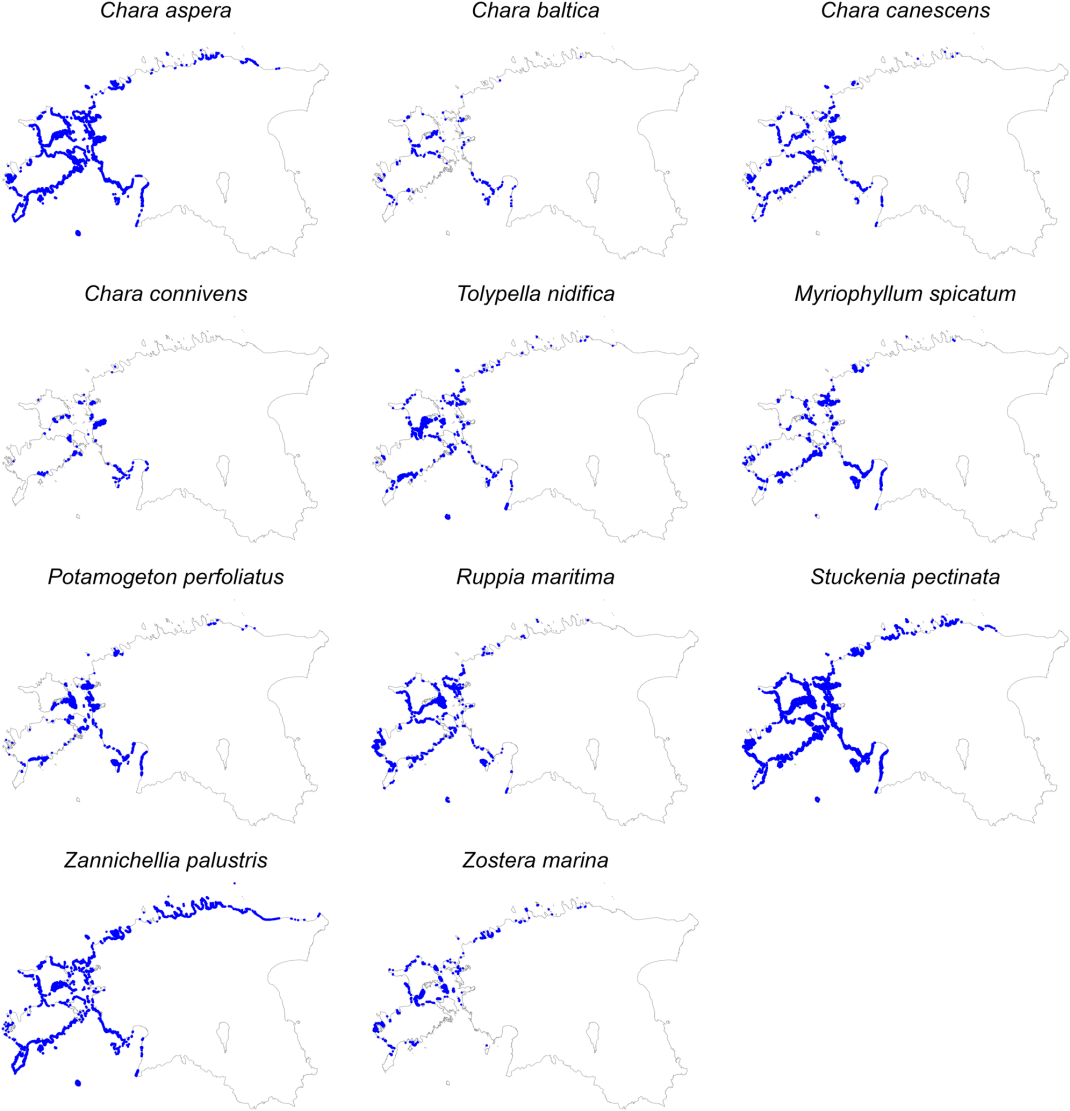
Modeled distributions of the studied species. Blue color represents the occurrence of a species. The size of the blue symbols of occurrence is increased compared to the original 100 × 100 m pixel size to improve readability. *R* programming language version 4.2.2 (https://www.r-project.org/) in the development environment *RStudio* (https://posit.co/products/open-source/rstudio/) was used to generate this map.

Based on the modeled species distributions, *S. pectinata* had by far the largest distribution area with 1125 km^2^. *C. aspera* ranked the second with 451 km^2^, all other species had distribution area below 400 km^2^. *C. baltica* had the smallest area with 26 km^2^ (Fig. 3). The majority of the species were spread along the whole coastline while *C. baltica* and *C. connivens* were mainly restricted to western Estonia. Unlike the other species, the presence of *Z. marina* in the Gulf of Riga was limited. Compared to angiosperms, the distribution of charophytes was limited to shallower areas (except for *T. nidifica*) and sheltered bays (Fig. 4).

SDM predictions showed that 29% of the modeling area was occupied by co-habitation of charophytes and angiosperms (i.e., at least one charophyte and one angiosperm present in a grid cell). 61% of the modeling area was occupied exclusively by angiosperms (i.e. at least one angiosperm present and no charophytes) while only 10% was occupied exclusively by charophytes (i.e. at least one charophyte present and no angiosperms).

**Figure 5:**
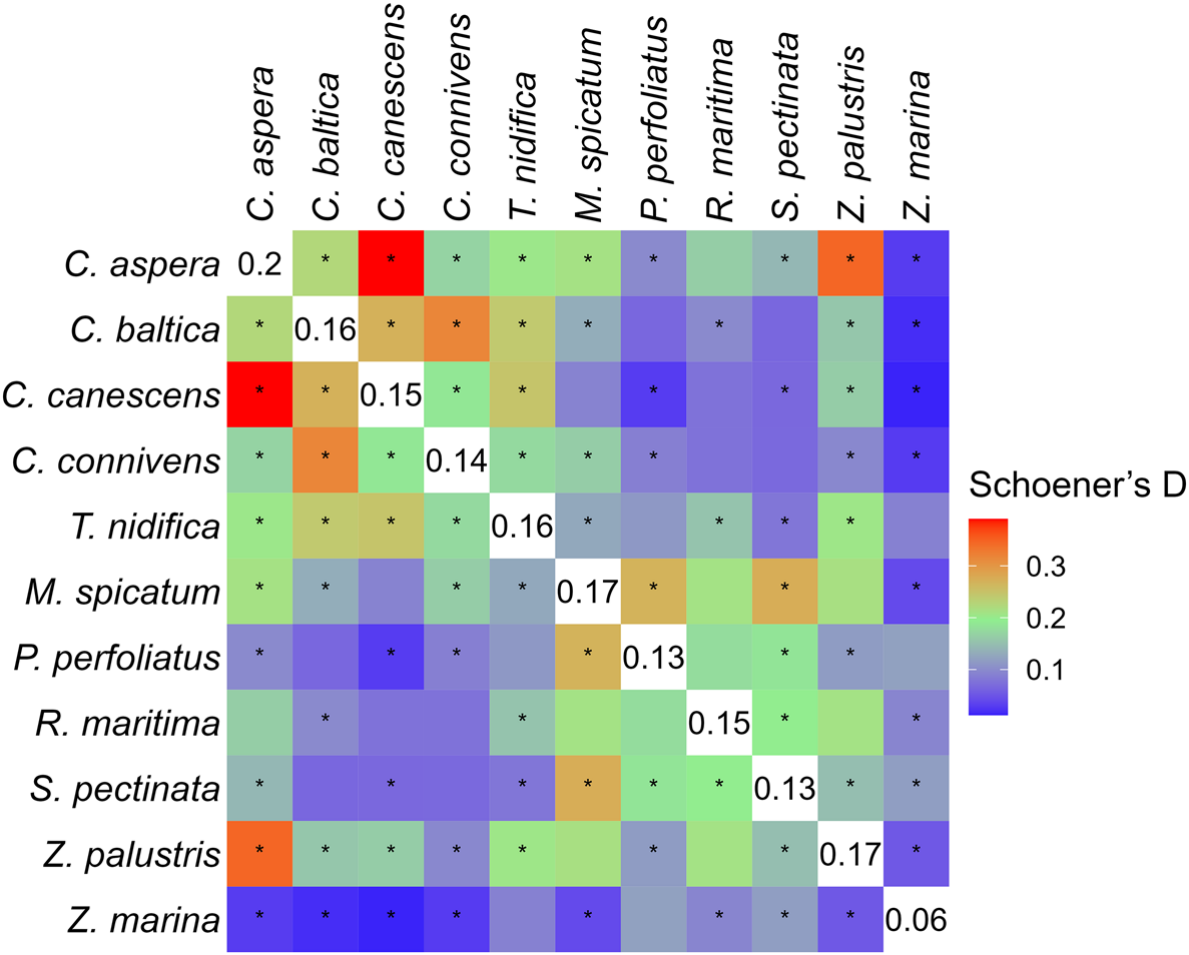
Occurrence overlap between the studied species based on field observations. Higher Schoener’s D value indicates larger overlap. The numbers in the diagonal represent average D values of the species. Asterisks indicate statistically significant D values (p ≤ 0.05, corrected using Benjamini–Hochberg method for multiple comparisons). A statistically significant value indicates that the observed D value differs from what would be expected by random chance.

Based on the field data, the highest distribution overlap was between *C. aspera* and *C. canescens* (Schoener’s D = 0.39) followed by *C. aspera* and *Z. palustris* (Schoener’s D = 0.35). *C. aspera* also had the largest mean overlap with all the other species. *Z. marina* had the smallest overlap with the other species (Fig. 5). Based on permutation tests, it was found that 41 out of the 55 unique pairs of species exhibited D values that were statistically different (p < 0.05) from what would be expected by random chance. The group-wise mean D values showed statistically significant differences among the species groups (Kruskal–Wallis test, p < 0.05). Specifically, the mean D value within charophyte species was the highest at 0.24, followed by a mean D value of 0.16 within angiosperms, and the mean D value between charophytes and angiosperms was the lowest at 0.11.

**Figure 6:**
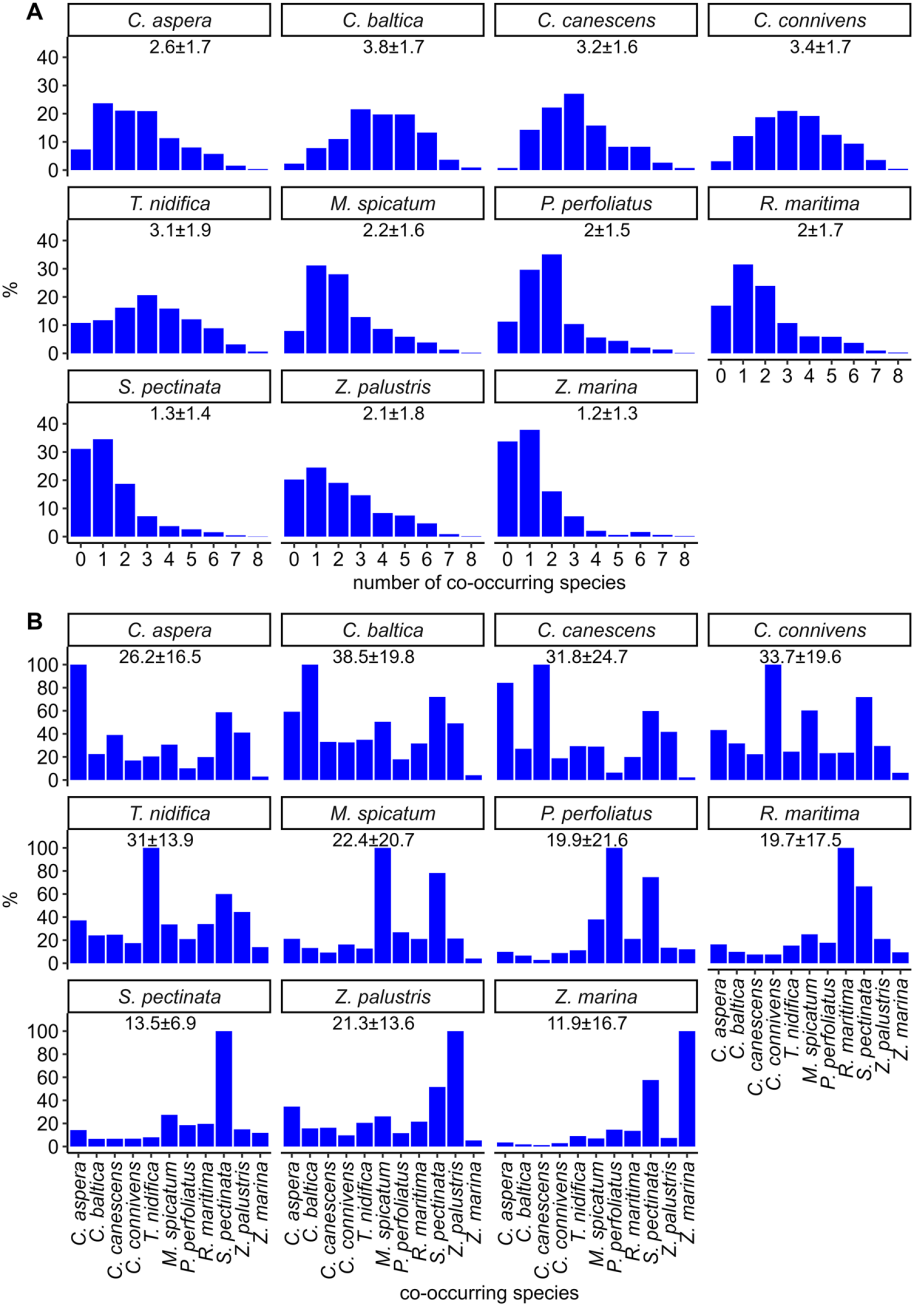
Proportions of occurrences with different number of co-occurring species (A) and proportions of occurrence overlap between the studied species (B) based on species’ field observations. The numeric values in each panel represent mean value ± standard deviation. For uniform x-axis among all plots, the focus species is also included with 100% value in panel B.

Charophytes *C. baltica*, *C. cansecens*, and *C. connivens* almost exclusively (> 99% of their distribution area) co-occurred with other species (Fig. 3). Most frequently the studied species had 2–3 co-occurring species (Fig. 6). Notable deviations from this pattern were *S. pectinata*, *Z. palustris* and *Z. marina* that most frequently had none or only one co-occurring species. The mean number of co-occurring species was the highest in *C. baltica*. The proportions of co-occurring in field sites were most evenly distributed in *S. pectinata* and *T. nidifica* while *S. pectinata* itself was the most common co-occurring species for all the other species (Fig. 6). *Z. marina* had the largest share of single-species occurrence (Fig. 3) and coexistence with charophytes was almost lacking (Figs. 5, 6).

## Discussion

The level of co-occurrence of the studied species varied along the environmental gradients (Fig. 2). This means that the potential rate of competition between the species depends on the environmental conditions. The highest rate of competition of the studied macrophytes can be expected in shallow depth, low wave exposure, soft seabed sediments and salinity around five (Fig. 2). According to previous studies, the distribution of charophytes and angiosperms is mainly controlled by depth that reflects light availability as well as hydrodynamic activity^19,98,99^. Likewise, depth was the most influential environmental variable in the distribution models of the studied macrophytes in this study. In the brackish Baltic Sea region, soft sediments are inhabited by charophytes and angiosperms of fresh, brackish, and marine water origin. Living close to their physiological limits amplifies the effect of abiotic environmental gradients and even small changes in salinity can cause significant changes in species’ distributions^100,101^. Most of the studied species can tolerate wider salinity range than the range present in the study area. However, *Z. marina*, *T. nidifica,* and *C. baltica* are more common in areas with salinity over 5. Eelgrass *Z. marina* also differs from the other species by preferring greater water depth^45,102^.

The most wide-spread soft-bottom species in the coastal waters of Estonia, northern Baltic Sea, was sago pondweed (*S. pectinata*) with an estimated distribution area of about 1100 km^2^. The species is globally widespread and is also one of the most common species in the brackish Baltic Sea region^25^. *S. pectinata* is of freshwater origin and can tolerate salinity fluxes up to 19^47^. The species is regarded as tolerant to eutrophication^103^ and has relatively wide niche space^45^. Angiosperms *Z. palustris*, *M. spicatum*, and *P. perfoliatus* and charophyte *C. aspera* had also large distribution area of about 300 km^2^ (Fig. 3, 4). Although regarded as relatively common species in the Estonian coastal waters and with relatively wide niche space^45^, the charophyte *C. baltica* was estimated to have the smallest distribution area of less than 50 km^2^. The salinity tolerance of *C. baltica* is probably the key factor: the species prefers salinity over two^104^ and does not penetrate the low-salinity estuaries which are common habitats for all the other studied charophyte species. *Z. palustris* and *Z. marina* have been shown to have the widest niche space followed by *C. aspera*, *T. nidifica,* and *S. pectinata* while *R. maritima*, *P. perfoliatus*, *M. spicatum,* and *C. connivens* had narrow niche space^45^. This shows that neither the numerous findings nor the large niche space directly translate into large distribution area in a given regional scale. Generally, realized distributions of generalist species tend to be wider than those of specialist species but there is a lot of variability and it cannot be considered as a strict rule^41,46,105,106^. Several earlier studies e.g. ^46,107–109^, support our findings about discrepancy between niche width and distribution area.

The aspect of abundance (biomass, percentage coverage, shoot density or other measure) was not addressed in this study but it must be noted that in addition to distribution area, the abundance is an important feature when estimating the status and viability of a species and need to be taken into account in marine spatial planning and nature conservation activities. In regard to variation in plant abundance, it has been shown in terrestrial environment (especially in agriculture) that an optimal density is required for healthy plants. It has also been shown in eelgrass that positive interactions among shoots in the early stage of colonization are more important than competitive processes^110^: increased plant density has positive effects on shoot growth, areal expansion of patches and rate of increase of shoot density. In the low-salinity areas of the Baltic Sea eelgrass reproduces asexually through growth of rhizomes that creates patches and meadows. In a multispecies angiosperm community, *Z. marina* has been shown to be more resistant to shading, but at the same time the monocultures of eelgrass showed faster recovery after disturbance^111^. However, the number of studies on interspecific interactions of submerged soft-bottom species in the Baltic Sea region is limited^111^ and no information exists for stress-tolerance of e.g., *T. nidifica* in mono- or multispecies communities. The revealed co-occurrence patterns of the studied species provide an important insight for the future targeted research on interspecific relationships.

The level of co-occurrence was higher inside the charophytes species group than inside the angiosperms species group. While the charophytes commonly co-exist with both charophytes and angiosperms, the angiosperms were more commonly associated with other angiosperms rather than charophytes. Moreover, three species of the *Chara* genus (*C. baltica*, *C. canescens*, *C. connivens*) showed very low proportion (less than 4%) of single-species occurrences while four angiosperm species out of six formed single-species communities in 17–34% of their field occurrence sites. Contrasting to our findings in the brackish water, formation of single-species meadows is noted to be characteristic of charophytes in freshwater^112^. *T. nidifica* differed from the other charophytes by exhibiting higher proportion of single-species occurrences and co-occurrence with *Z. marina* (Fig. 6). *T. nidifica* is found in low coverage both within charophyte and vascular plant communities, however, it is also often found as a single specimen on bare sand. It is a brackish water species and grows mostly in salinities over four, but can tolerate large range of salinity^21,113^. In Estonia the species is found growing in depth from 0.1 to 9.5 m, but it is most common in shallower area with depth range 1–3 m^45^.

The highest level of co-occurrence, as measured by the Schoener’s D, was observed between *C. aspera* and *C. canescens* (Fig. 5). However, the overlap was not symmetrical: 84% of the field occurrences of *C. canescens* overlapped with that of *C. aspera* but only 39% of the occurrences of *C. aspera* coincided with that of *C. canescens* (Fig. 6). The distribution area of *C. aspera* was more than twice the size of that of *C. canescens*. While both species are widespread in the Baltic Sea, it has been shown that the difference between them is that *C. aspera* often forms monospecific stands or dominant communities with association of other species while *C. canescens* mainly occurs as single plants or patches inside the stands of *C. aspera* or other charophytes^21^.

*Z. marina* had the lowest co-occurrence with all other species; the overlap was particularly low with charophytes (Fig. 5). The rare co-occurrence of *Z. marina* and the *Chara* genus of charophytes is an anticipated result, but in this study, the pattern was quantified using extensive database of field observations together with permutation tests. Eelgrass is a marine species and cannot tolerate salinity lower than five. In Estonian coastal waters, *Z. marina* commonly forms single-species communities in a depth range of 2–5 m and overall, it has been found growing in depths from 0.1 to 8.6 m. Our results also clearly indicated that compared to the other studied species, *Z. marina* had the largest proportion of single-species occurrences. When *Z. marina* grew with other species, then it had the highest proportion of overlap (58%) with the species with the largest distribution area—*S. pectinata*. While the overlap with the species of genus *Chara* was very low (< 4%), the occurrence of *Z. marina* overlapped with that of the charophyte *T. nidifica* by almost 10% (Fig. 6).

*Potamogeton perfoliatus* was another angiosperm species that had notably low occurrence overlap with the species of *Chara* genus. While in the case of *Z. marina* and *Chara* spp., the lack of overlap can be clearly explained by variations in depth, salinity and wave exposure, the segregation of *P. perfoliatus* and *Chara* spp. is not that straightforward. It seems that for *P. perfoliatus*, the sediment composition has the most important role—the distribution of *P. perfoliatus* is limited to soft sediments with the share of sand around 70–100% while the share of sand can be much smaller in case of the species of *Chara* genus^45^.

It has been previously shown that among the studied species, the charophyte *C. connivens* and angiosperm *M. spicatum* grow in the most similar environmental conditions^45^. Although there is significant overlap in the distribution area of these species in the NE Baltic Sea region, *M. spicatum* had more than two-fold larger distribution area than that of *C. connivens* because *M. spicatum* also inhabits more exposed coasts. However, both species occur more commonly together with *S. pectinata* than with each other. This indicates that similar niches do not necessarily translate into highest distribution overlap but instead, the highest distribution overlap exists with the most widespread species. Theoretically, there are two extreme scenarios in the niche–distribution interplay^114–116^: if competitive interactions strongly determine community composition, then the species with similar niches should be spatially segregated; if community composition is not strongly driven by competition, then the species with similar niches should exhibit high spatial overlap. Based on empirical evidence of different organism groups e.g. ^114,117^, and references therein, the relationships between niche overlap and spatial overlap can take various forms. Based on the results of this study and previous studies^22^, frequent co-occurrence of the studied species existed and thus it is more likely that the competition is not very important structuring force of communities of soft-bottom macrophytes in the northern Baltic Sea. However, it must be considered that spatial and temporal scales play an important role in the outcome of distribution studies. Closely related species or species with similar niches rely on mutually exclusive distribution at small spatial scales due to competition but co-occur in larger spatial scales due to similar broad-scale environmental preferences^51,118,119^. This means that distribution studies should address multiple spatial scales (from microhabitat to regional level) in order to fully understand the coexistence and competition patterns between species. In this study, the environmental variables were available in 10–50 m raster layers. We lacked the small-scale (sub-meter to below 10 m) environmental data were direct competition between species actually takes place. Such high resolution environmental data is lacking for large marine areas due to obvious practical reasons. The coexistence patterns of soft bottom macrophytes in small spatial scales remain to be elucidated in dedicated future field studies.

Species’ potential geographic distribution areas often differ from their occupied distribution areas due to biotic interactions^117^. The complex interplay between plants’ physiological tolerance limits, availability of environmental niche and biotic interactions (competition, herbivory, pathogens etc.) is further modified by stochastic disturbance events (e.g., severe storms) and anthropogenic pressures (e.g., eutrophication, human-induced climate change). The complexity is further increased by concurrent effects and interactions acting at different spatial and temporal scales. Thus, it must be taken into account that results of SDMs only provide simplified insights into geographic aspect of the species coexistence but cannot reveal the full complexity of the system.

## Conclusions

Analyzing a comprehensive dataset of field samples of soft substrate macrophytes, we found that the observed co-occurrence patterns among sympatric species significantly diverged from what would be expected by random chance. The analysis showed that species exhibited a higher tendency to co-occur within their taxonomic groups (charophytes and angiosperms) than between these groups. When considering previous studies of niche positions and niche sizes of the studied species, it can be concluded that large niche space does not always directly translate into large distribution area in a given regional scale. In future studies multiple spatial scales (from microhabitat to regional level) as well as temporal scales should be addressed to fully understand the coexistence and competition patterns between sympatric macrophyte species.

### Supplementary Information

Supplementary Information 1.

Supplementary Information 2.

Supplementary Information 3.

## Data Availability

Enquiries about data availability should be directed to Kristjan Herkül (kristjan.herkul@ut.ee).

## Code availability

Custom R code, deemed central to the conclusions, is added in Appendix 2.
