# Supplementary material for: Distribution and co-occurrence patterns of charophytes and angiosperms in the northern Baltic Sea
Source: Sci Rep. 2023 Nov 16;13:20096. doi: 10.1038/s41598-023-47176-8 (PMC10654418; doi:10.1038/s41598-023-47176-8)

**Appendix 1. Schematic overview maps of environmental variables.** Full names of the variables and additional information is in Table 2. Values of all variables are scaled between zero and one in order to display the general spatial patterns of the variables on the same color scale. Estonian coasline and border is shown with gray line. *R* programming language version 4.2.2 (https://www.r-project.org/) in the development environment *RStudio* (https://posit.co/products/open-source/rstudio/) was used to generate this map.


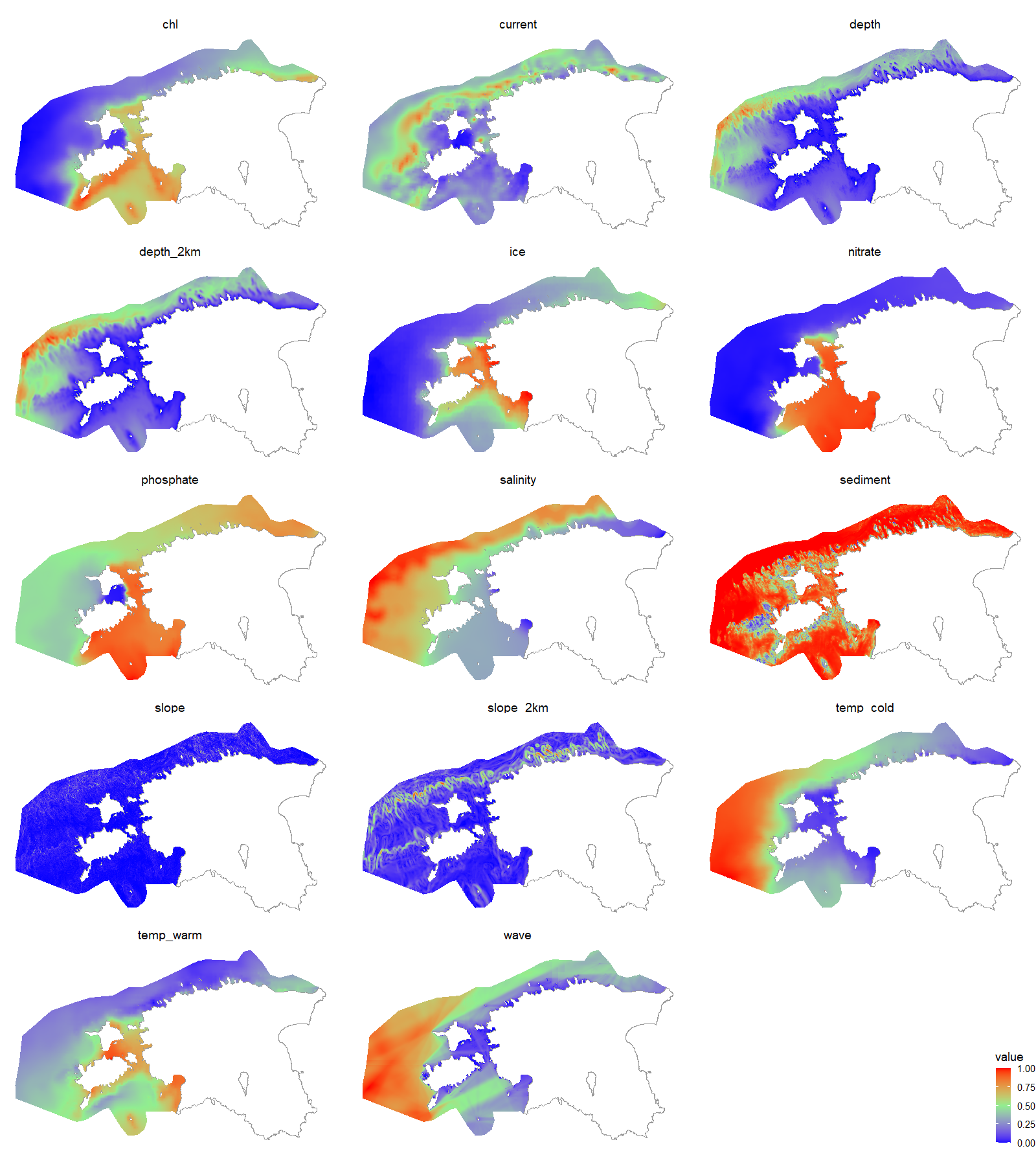

Supplement: Supplementary file 1 — Supplementary Information 1. [file 41598_2023_47176_MOESM1_ESM.docx]
