# Supplementary material for: Distribution and co-occurrence patterns of charophytes and angiosperms in the northern Baltic Sea
Source: Sci Rep. 2023 Nov 16;13:20096. doi: 10.1038/s41598-023-47176-8 (PMC10654418; doi:10.1038/s41598-023-47176-8)

# Appendix 2. R code of random forest modeling and co-occurrence analysis (Schoener's D)

## Load packages

```
library(future.apply)
library(caret)
library(randomForest)
library(PresenceAbsence)
library(pROC)
library(tidyverse);options(dplyr.width = Inf)
```

## Load data

### Training data

Rows are sites, occurrence of macrophyte species (0 - absence, 1 - presence) and values of environmental variables are in columns.

```
train <- readRDS('train.rds')
str(train)
```

```
## tibble [14,356 x 26] (S3: tbl_df/tbl/data.frame)
##  $ site_id                : num [1:14356] 11682 203938 293772 736485 991332 ...
##  $ Chara_aspera           : num [1:14356] 0 0 0 0 0 0 0 0 0 0 ...
##  $ Chara_baltica          : num [1:14356] 0 0 0 0 0 0 0 0 0 0 ...
##  $ Chara_canescens        : num [1:14356] 0 0 0 0 0 0 0 0 0 0 ...
##  $ Chara_connivens        : num [1:14356] 0 0 0 0 0 0 0 0 0 0 ...
##  $ Tolypella_nidifica     : num [1:14356] 0 0 0 0 0 0 0 0 0 0 ...
##  $ Myriophyllum_spicatum  : num [1:14356] 0 0 0 0 0 0 0 0 0 0 ...
##  $ Potamogeton_perfoliatus: num [1:14356] 0 0 0 0 0 0 0 0 0 0 ...
##  $ Ruppia_maritima        : num [1:14356] 0 0 0 0 0 0 0 0 0 0 ...
##  $ Stuckenia_pectinata    : num [1:14356] 0 0 0 0 0 0 0 0 0 0 ...
##  $ Zannichellia_palustris : num [1:14356] 0 0 0 0 0 0 0 0 0 0 ...
##  $ Zostera_marina         : num [1:14356] 0 0 0 0 0 0 0 0 0 0 ...
##  $ chl                    : num [1:14356] 2.6 2.6 2.61 2.53 2.69 ...
##  $ current                : num [1:14356] 0.0256 0.0265 0.0278 0.0297 0.0309 ...
##  $ depth                  : num [1:14356] 67.9 80.1 72.5 81.5 67.2 ...
##  $ depth_2km              : num [1:14356] 63.9 72.7 68.7 77.8 63.1 ...
##  $ slope                  : num [1:14356] 0.322 0.855 0.285 0.524 0.288 ...
##  $ slope_2km              : num [1:14356] 0.189 0.253 0.622 0.103 0.399 ...
##  $ wave                   : num [1:14356] 382292 347466 346184 357414 350525 ...
##  $ ice                    : num [1:14356] 0.286 0.28 0.28 0.257 0.278 ...
##  $ sediment               : num [1:14356] 0.983 0.958 0.961 0.966 0.984 ...
##  $ nitrate                : num [1:14356] 6.23 6.06 6 5.53 5.89 ...
##  $ phosphate              : num [1:14356] 1.008 0.993 0.988 0.955 0.98 ...
##  $ salinity               : num [1:14356] 8.44 8.28 8.2 8.47 7.16 ...
##  $ temp_cold              : num [1:14356] 2.71 2.74 2.74 2.85 2.74 ...
##  $ temp_warm              : num [1:14356] 13.4 13.5 13.5 13.3 13.6 ...
```

### Prediction data

Rows are grid points in the prediction grid with 100 m step, values of environmental variables are in columns.

```
pred <- readRDS('pred.rds')
str(pred)
```

```
## tibble [3,685,338 x 17] (S3: tbl_df/tbl/data.frame)
##  $ grid_id  : int [1:3685338] 1 3 5 21 23 25 27 29 31 33 ...
##  $ x        : num [1:3685338] 630359 630459 630559 629859 629959 ...
##  $ y        : num [1:3685338] 6653291 6653291 6653291 6653191 6653191 ...
##  $ chl      : num [1:3685338] 2.6 2.6 2.6 2.6 2.6 ...
##  $ current  : num [1:3685338] 0.0256 0.0256 0.0256 0.0256 0.0256 ...
##  $ depth    : num [1:3685338] 64.1 64.4 64.3 62.7 63.1 ...
##  $ depth_2km: num [1:3685338] 63.1 63.2 63.2 63.4 63.3 ...
##  $ slope    : num [1:3685338] 0.2 0.215 0.39 0.248 0.352 ...
##  $ slope_2km: num [1:3685338] 0.0165 0.0259 0.0425 0.0567 0.053 ...
##  $ wave     : num [1:3685338] 398452 398257 398051 398524 398386 ...
##  $ ice      : num [1:3685338] 0.287 0.287 0.287 0.287 0.287 ...
##  $ sediment : num [1:3685338] 0.98 0.982 0.982 0.985 0.985 ...
##  $ nitrate  : num [1:3685338] 6.22 6.23 6.23 6.22 6.22 ...
##  $ phosphate: num [1:3685338] 1.01 1.01 1.01 1.01 1.01 ...
##  $ salinity : num [1:3685338] 8.47 8.46 8.46 8.48 8.48 ...
##  $ temp_cold: num [1:3685338] 2.71 2.71 2.71 2.71 2.71 ...
##  $ temp_warm: num [1:3685338] 13.4 13.4 13.4 13.4 13.4 ...
```

## Models and spatial predictions

Vector of species names

```
species <- names(train)[2:12]
print(species)
```

```
##  [1] "Chara_aspera"             "Chara_baltica"
##  [3] "Chara_canescens"          "Chara_connivens"
##  [5] "Tolypella_nidifica"       "Myriophyllum_spicatum"
##  [7] "Potamogeton_perfoliatus" "Ruppia_maritima"
##  [9] "Stuckenia_pectinata"      "Zannichellia_palustris"
## [11] "Zostera_marina"
```

### Fit regression-type RF models

Train models for each species

```
plan(multisession)
set.seed(101)
models.reg <- future_lapply(species, function(i) {
  randomForest(
    get(i) ~ chl + current + depth + depth_2km + slope + slope_2km + wave + ice +
      sediment + nitrate + phosphate + salinity + temp_cold + temp_warm,
    ntree = 1000, data = train, importance = T, nPerm = 10
  )
})
plan(sequential)
names(models.reg) <- species
```

Make predictions of each species for each data point in the prediction dataset

```
plan(multisession)
predictions.reg <- bind_cols(
```

```
  pred %>% select(x, y),
  future_lapply(models.reg, function(i)
    predict(i, newdata = pred, type = 'response')) %>% as_tibble
)
plan(sequential)
```

Convert predicted probabilities of occurrence to binary presence-absence using the sensitivity-specificity difference minimizer method

```
# Generate predictions for input data
predictions.reg.input <- lapply(models.reg, function(i)
  predict(i, newdata = train, type = 'response')) %>% as_tibble

# Calculate thresholds for probability of occurrence
thresholds <- sapply(species, function(i) {
  optimal.thresholds(
    cbind(train['site_id'], train[i], predictions.reg.input[i]),
    opt.methods = 'Sens=Spec'
  )[1,2]
}) %>% as_tibble(rownames = 'species')

# Based on the thresholds, convert probability of occurrence to presence-absence and
# add the presence-absence columns to the predictions table
for (i in species) {
  t <- thresholds$value[thresholds$species == i]
  predictions.reg[, paste0(i, '.pa')] <- ifelse(predictions.reg[, i] >= t, 1, 0)
}
```

Calculate species distribution areas in square kilometers

```
areas <- tibble(
  species = species,
  area_km2 = predictions.reg %>% select(matches('\\.pa$')) %>%
    colSums * (100 * 100) / 1000000
)
```

### Fit classification-type RF models

Convert species numeric occurrences to factors

```
train.class <- train %>% mutate(across(any_of(species), factor))
```

Train models for each species

```
plan(multisession)
set.seed(101)
models.class <- future_lapply(species, function(i) {
  randomForest(
    get(i) ~ chl + current + depth + depth_2km + slope + slope_2km + wave + ice +
      sediment + nitrate + phosphate + salinity + temp_cold + temp_warm,
    ntree = 1000, data = train.class, importance = T, nPerm = 10
  )
})
plan(sequential)
names(models.class) <- species
```

Make predictions of each species for each data point in the prediction dataset

```r
plan(multisession)
predictions.class <- bind_cols(
  pred %>% select(x, y),
  future_lapply(models.class, function(i)
    predict(i, newdata = pred, type = 'response')) %>% as_tibble
)
plan(sequential)
```

### 10-fold cross-validation of RF models

Create folds for each species

```r
set.seed(101)
cv.folds <- sapply(species, function(i)
  createFolds(factor(train[[i]]), k = 10, list = T), simplify = F, USE.NAMES = T)
```

Calculate cross-validation statistics for regression-type RF

```r
# calculate statistics, store values in list
plan(multisession)
cv.reg <- future_lapply(species, function(i) {
  sapply (1:10, function(f) {
    d.train <- train %>% slice(cv.folds[[i]][-f] %>% reduce(c))
    d.test <- train %>% slice(cv.folds[[i]][[f]])
    mdl <- randomForest(
      get(i) ~ chl + current + depth + depth_2km + slope + slope_2km + wave + ice +
        sediment + nitrate + phosphate + salinity + temp_cold + temp_warm,
      ntree = 1000, data = d.train)
    observed <- train %>% slice(cv.folds[[i]][[f]]) %>% .[[i]]
    predicted.test <- predict(mdl, newdata = d.test)
    predicted.train <- predict(mdl, newdata = d.train)
    pa.thr <- optimal.thresholds(
      cbind(d.train['site_id'], d.train[i], predicted.train),
      opt.methods = 'Sens=Spec'
    )[1,2]
    auc <- pROC::auc(observed, predicted.test)
    predicted.test.pa <- factor(ifelse(predicted.test >= pa.thr, 1, 0))
    observed <- factor(observed)
    cm <- caret::confusionMatrix(predicted.test.pa, observed)
    kappa <- cm$overall['Kappa']
    cbind(auc, kappa)
  })
}, future.seed = 101)
plan(sequential)

# from list to table
cv.reg <- lapply(1:length(species), function(i) {
  cv.reg[[i]] %>% t %>% as.data.frame %>% setNames(c('auc', 'kappa')) %>%
    tibble %>% mutate(species = species[i]) %>% relocate(species)
  }) %>% bind_rows
```

Mean statistics over all folds for each species

```r
cv.reg %>% group_by(species) %>% summarise(across(c(auc:kappa), ~ round(mean(.), 3)))
```

```
## # A tibble: 11 x 3
```

```
##    species                    auc kappa
##    <chr>                    <dbl> <dbl>
##  1 Chara_aspera             0.963 0.529
##  2 Chara_baltica            0.943 0.274
##  3 Chara_canescens          0.95  0.36
##  4 Chara_connivens          0.947 0.275
##  5 Myriophyllum_spicatum    0.948 0.507
##  6 Potamogeton_perfoliatus  0.955 0.462
##  7 Ruppia_maritima          0.926 0.348
##  8 Stuckenia_pectinata      0.948 0.645
##  9 Tolypella_nidifica       0.894 0.156
## 10 Zannichellia_palustris   0.901 0.323
## 11 Zostera_marina           0.943 0.427
```

Calculate cross-validation statistics for classification-type RF

```
# calculate statistics, store values in list
plan(multisession)
cv.class <- future_lapply(species, function(i) {
  sapply (1:10, function(f) {
    d.train <- train.class %>% slice(cv.folds[[i]][-f] %>% reduce(c))
    d.test <- train.class %>% slice(cv.folds[[i]][[f]])
    mdl <- randomForest(
      get(i) ~ chl + current + depth + depth_2km + slope + slope_2km + wave + ice +
        sediment + nitrate + phosphate + salinity + temp_cold + temp_warm,
      ntree = 1000, data = d.train)
    observed <- train.class %>% slice(cv.folds[[i]][[f]]) %>% .[[i]]
    predicted.test <- predict(mdl, newdata = d.test)
    cm <- caret::confusionMatrix(predicted.test, observed)
    kappa <- cm$overall['Kappa']
    cbind(kappa)
  })
}, future.seed = 101)
plan(sequential)

# from list to table
cv.class <- lapply(1:length(species), function(i) {
  cv.class[[i]] %>% as.data.frame %>% setNames(c('kappa')) %>%
    tibble %>% mutate(species = species[i]) %>% relocate(species)
  }) %>% bind_rows
```

Mean statistics over all folds for each species

```
cv.class %>% group_by(species) %>% summarise(across(c(kappa), ~ round(mean(.), 3)))
```

```
## # A tibble: 11 x 2
##    species                  kappa
##    <chr>                    <dbl>
##  1 Chara_aspera             0.498
##  2 Chara_baltica            0.213
##  3 Chara_canescens          0.288
##  4 Chara_connivens          0.236
##  5 Myriophyllum_spicatum    0.469
##  6 Potamogeton_perfoliatus  0.424
##  7 Ruppia_maritima          0.309
##  8 Stuckenia_pectinata      0.632
```

```
##   9 Tolypella_nidifica      0.127
## 10 Zannichellia_palustris  0.285
## 11 Zostera_marina          0.351
```

## Co-occurrence analysis

Function for calculating Schoener's D index

```
# arguments spe1 and spe2 are the vectors of two species
# function returns the D index of the two species
schoe <- function(spe1, spe2) {
  px = spe1 / sum(spe1)
  py = spe2 / sum(spe2)
  D = 1 - (0.5 * sum(abs(px - py)))
  return(D)
}
# testing the function
schoe(
  c(0,0,0,0,0,1,1,1,1,1),
  c(1,1,1,1,1,0,0,0,0,0)
)
```

```
## [1] 0
```

```
schoe(
  c(1,1,1,1,1,1,1,1,1,1),
  c(1,1,1,1,1,0,0,0,0,0)
)
```

```
## [1] 0.5
```

```
# D not affected by an excess of empty samples
schoe(
  c(1,1,1,1,1,0,0,0,0,0),
  c(1,1,1,1,1,0,0,0,0,0)
)
```

```
## [1] 1
```

```
schoe(
  c(1,1,1,1,1),
  c(1,1,1,1,1)
)
```

```
## [1] 1
```

Calculate Schoener's D for every pair of species. Schoener's D is not sensitive to empty samples but empty samples must be removed for permutation test to avoid random combinations with samples from deep water outside potential range of the studied macrophytes

```
# generate unique 2-way combinations of species
cooc <- combn(species, 2) %>% t %>% as.data.frame %>% as_tibble %>%
  setNames(c('spe1', 'spe2'))
# include only sites with at least one species present
train.d <- train %>% select(any_of(species)) %>% mutate(sum = rowSums(.)) %>%
  filter(sum > 0) %>% select(-sum)
# calculate D for all pairs
cooc$d <- sapply(1:nrow(cooc), function(i)
```

```
  schoe(train.d[cooc$spe1[i]], train.d[cooc$spe2[i]])
)
```

Using permutations (100 000), test if the observed D value is significantly different from random

```
# generate permuted D values by randomly shuffling sites (rows);
# this produces null distribution expected by random chance
n <- nrow(train.d)
nperm <- 100000
plan(multisession)
d.perm <- future_lapply(1:nrow(cooc), function(i) {
  replicate(nperm,
            schoe(
              train.d[sample(1:n, n), cooc$spe1[i]],
              train.d[sample(1:n, n), cooc$spe2[i]]
            )
  )
}, future.seed = TRUE)
plan(sequential)

# mean permutational D
cooc$d.perm <- sapply(d.perm, mean)
# p-values: a proportion of permutational D values more extreme than the observed D value
cooc$p <- sapply(1:nrow(cooc), function(i) {
  observed <- cooc$d[i]
  permuted <- d.perm[[i]]
  permuted.mean <- mean(permuted)
  if (observed >= permuted.mean) {
    ((sum(permuted >= observed) + 1) / (length(permuted) + 1)) * 2
  } else {
    ((sum(permuted < observed) + 1) / (length(permuted) + 1)) * 2
  }
})
# corrected p-values for multiple comparisons (Benjamini-Hochberg procedure)
cooc$p.bh <- p.adjust(cooc$p, method = "BH")
# mark significant (p <= 0.05) pairs with asterisk for plotting
cooc$signif.bh <- ifelse(cooc$p.bh <= 0.05, '*', '')
```

Visualize some contrasting permutation test results: histogram of permuted D values shown in grey bars, blue line shows the observed D value

```
i = 4
ggplot(data.frame(permuted_D = d.perm[[i]]), aes(permuted_D)) +
  geom_histogram() +
  geom_vline(xintercept = cooc$d[i], color = 'blue', lwd = 1) +
  labs(
    title = paste0(cooc$spe1[i], ' vs ', cooc$spe2[i]),
    subtitle = 'observed D larger than expected by random chance (p < 0.05)'
  )
```

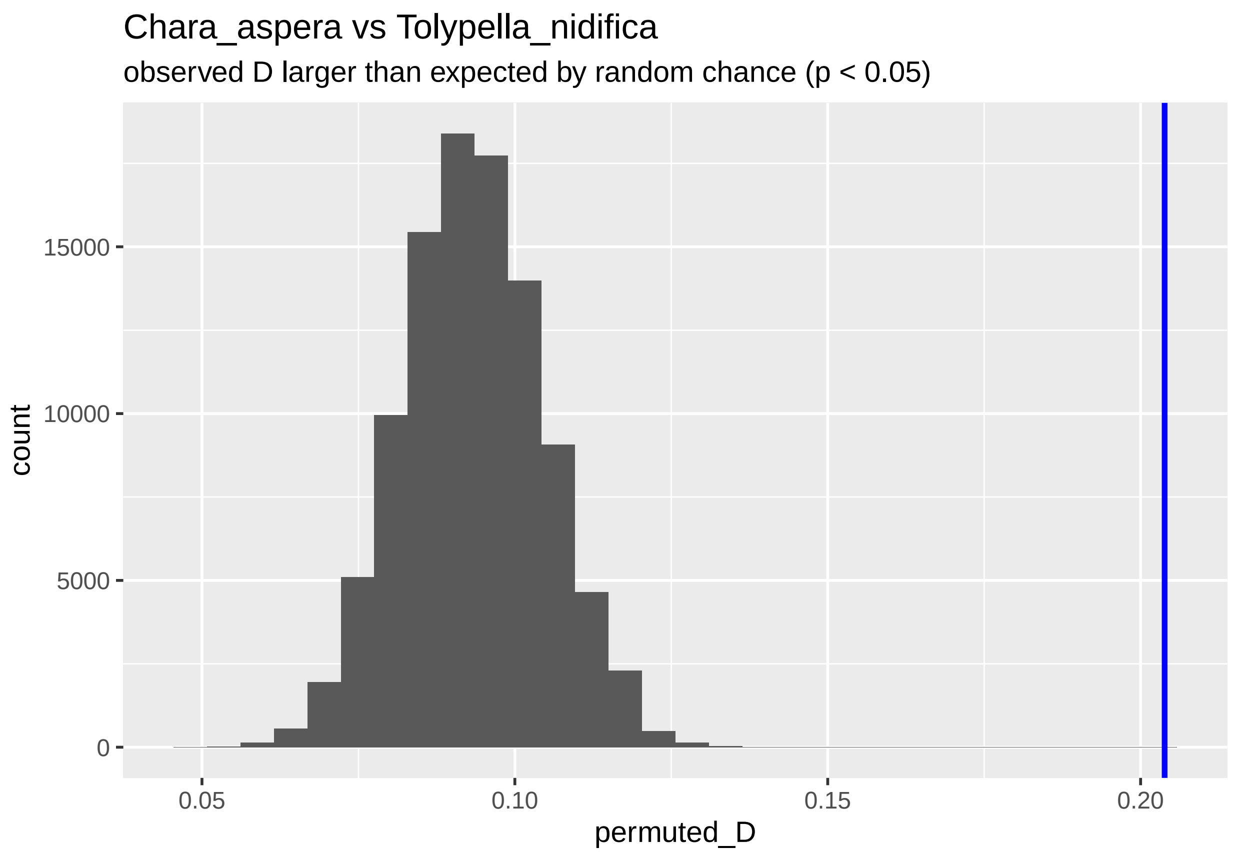


```r
i = 23
ggplot(data.frame(permuted_D = d.perm[[i]]), aes(permuted_D)) +
  geom_histogram() +
  geom_vline(xintercept = cooc$d[i], color = 'blue', lwd = 1) +
  labs(
    title = paste0(cooc$spe1[i], ' vs ', cooc$spe2[i]),
    subtitle = 'observed D smaller than expected by random chance (p < 0.05)')
```

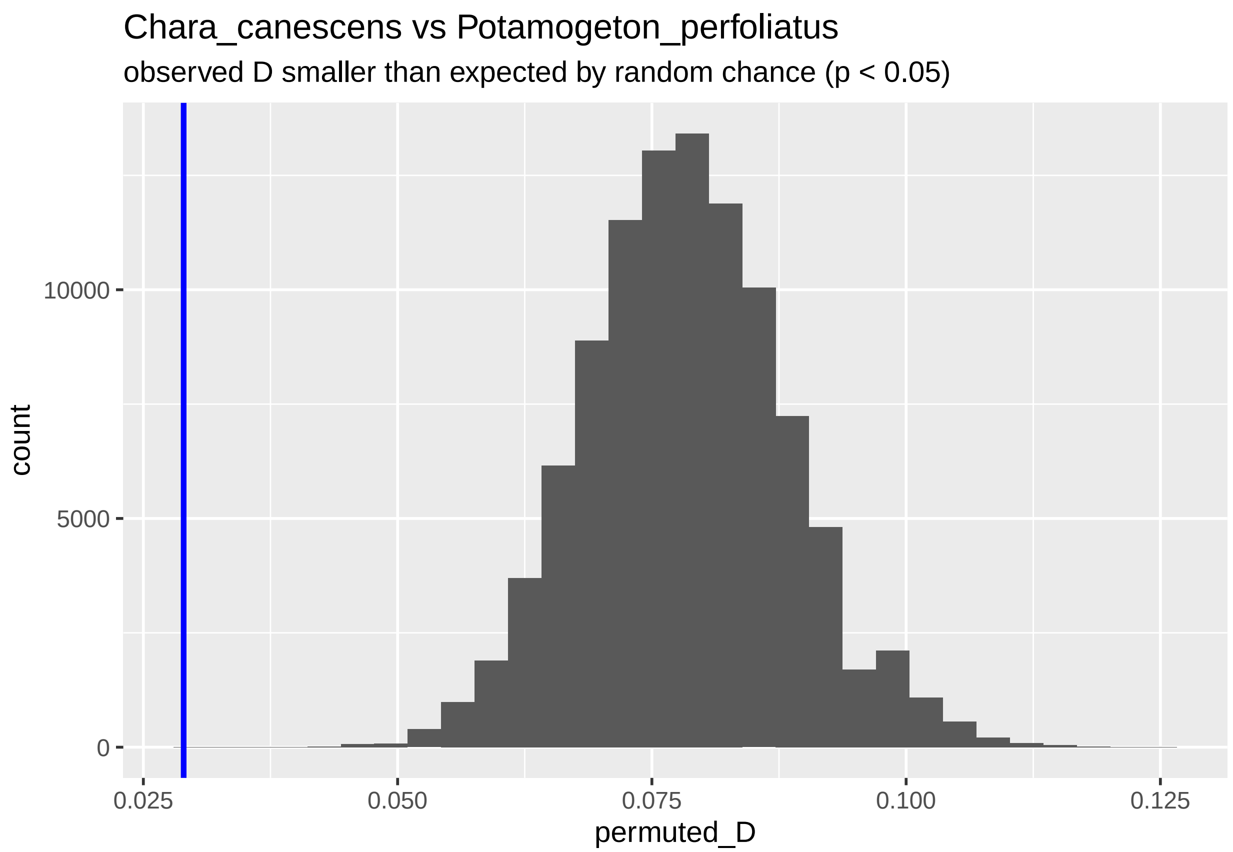


```
i = 17
ggplot(data.frame(permuted_D = d.perm[[i]]), aes(permuted_D)) +
  geom_histogram() +
  geom_vline(xintercept = cooc$d[i], color = 'blue', lwd = 1) +
  labs(
    title = paste0(cooc$spe1[i], ' vs ', cooc$spe2[i]),
    subtitle = 'observed D is not extreme compared to null distribution (p > 0.05)')
```

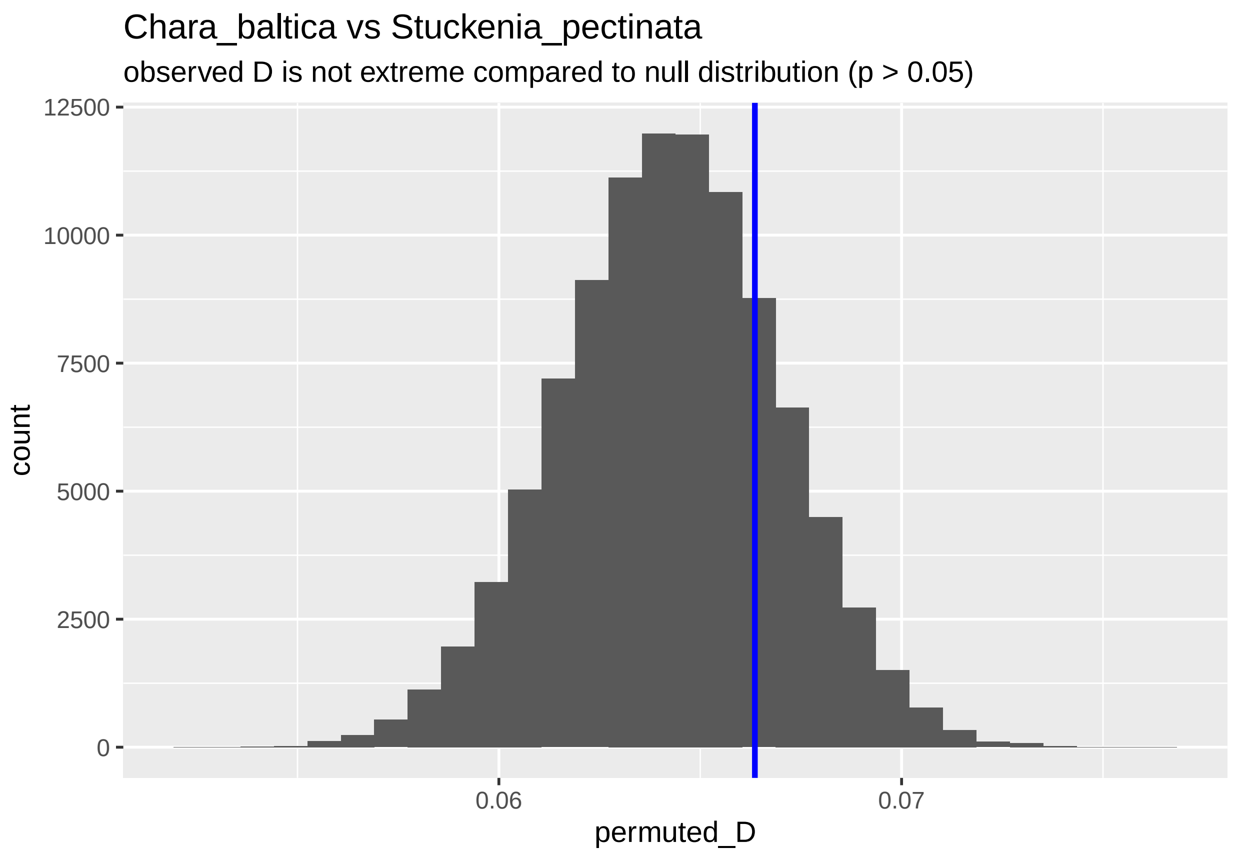


Generate plot of pairwise D values

```
# generate full matrix and factorize species names for plotting
cooc <- bind_rows(
  cooc,
  cooc %>% rename(spe1 = spe2, spe2 = spe1)
)
cooc$spe1 <- factor(cooc$spe1, species)
cooc$spe2 <- factor(cooc$spe2, rev(species))
# calculate mean D value for each species
d.spe <- cooc %>% group_by(spe1) %>% summarise(d = round(mean(d), 2)) %>% pull(d)
```

```
# plot
ggplot(cooc, aes(spe1, spe2, fill = d)) +
  theme_void(base_size = 16) +
  theme(
    axis.title = element_blank(),
    axis.text = element_text(face = 'italic'),
    axis.text.x = element_text(angle = 90, hjust = 0, vjust = 0.5),
    axis.text.y = element_text(hjust = 1, vjust = 0.5)
  ) +
  scale_x_discrete(position = 'top') +
  coord_fixed(ratio = 1) +
  geom_tile() +
  scale_fill_gradient2(low = "blue", mid = "lightgreen", high = "red",
                       midpoint = 0.195, na.value = "white", name = 'Schoener's D') +
  annotate('text', x = 1:11, y = 11:1, label = d.spe, size = 5) +
```

```
geom_text(aes(label = signif.bh))
```

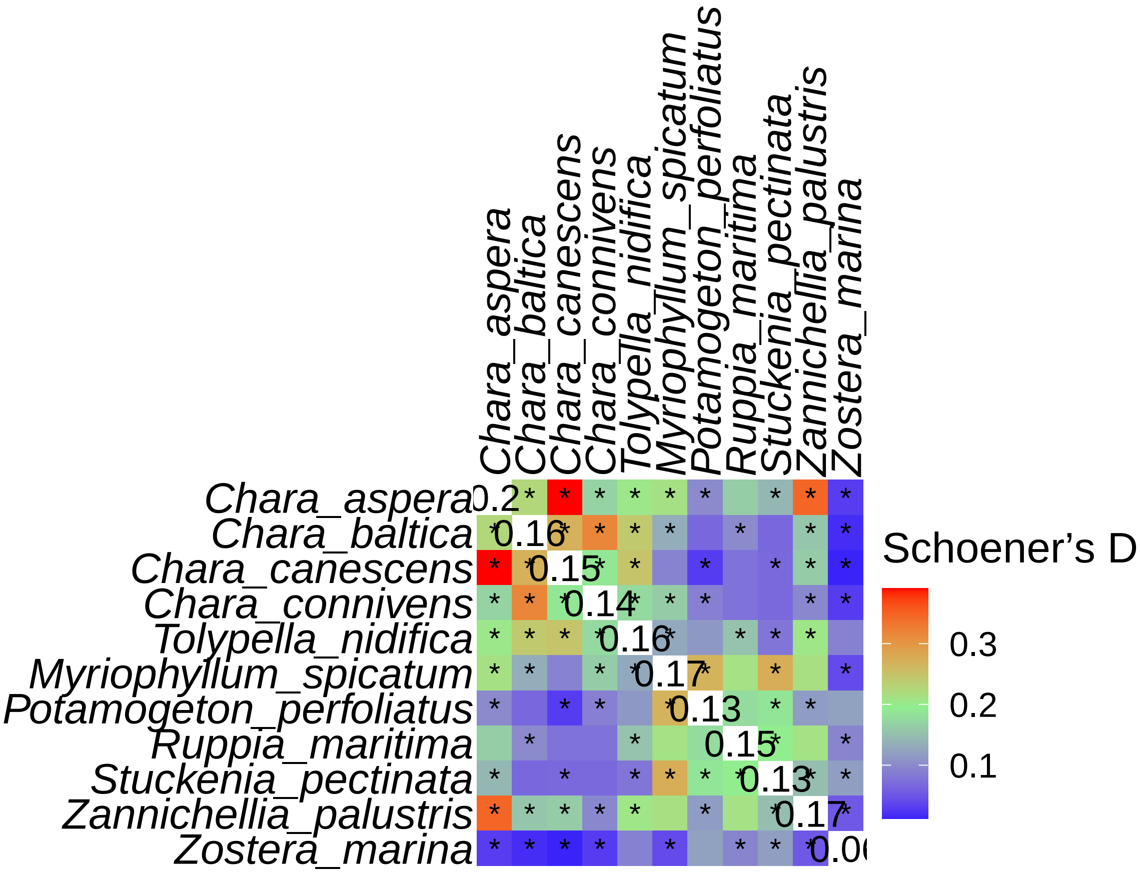

Supplement: Supplementary file 2 — Supplementary Information 2. [file 41598_2023_47176_MOESM2_ESM.pdf]
