# Supplementary material for: Distribution and co-occurrence patterns of charophytes and angiosperms in the northern Baltic Sea
Source: Sci Rep. 2023 Nov 16;13:20096. doi: 10.1038/s41598-023-47176-8 (PMC10654418; doi:10.1038/s41598-023-47176-8)

**Appendix 3. Importance of the environmental variables in predicting the distribution of the studied macrophyte species in the regression-type random forest models.** Higher value indicates higher importance. The full names or environmental variables are shown in Table 2.


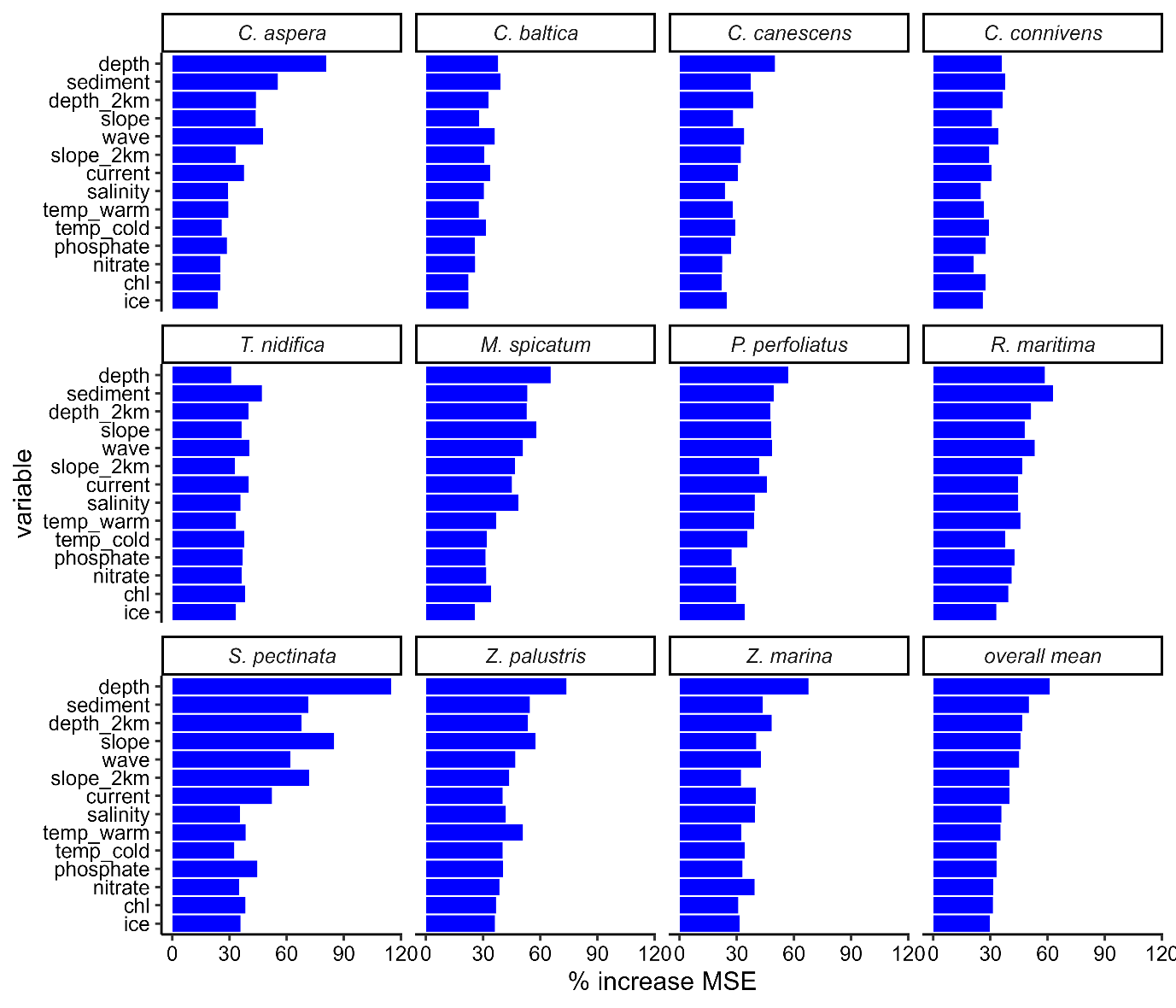

Supplement: Supplementary file 3 — Supplementary Information 3. [file 41598_2023_47176_MOESM3_ESM.docx]
